# Oral HIV pre-exposure prophylaxis use among pregnant and postpartum women: results from real-world implementation in Lesotho

**DOI:** 10.3389/frph.2023.1221752

**Published:** 2023-07-31

**Authors:** Lieketseng J. Masenyetse, Lauren Greenberg, Felleng Samonyane, Bokang Sekepe, Majoalane Mokone, Mafusi J. Mokone, Vincent J. Tukei, Laura K. Beres

**Affiliations:** ^1^ Elizabeth Glaser Pediatric AIDS Foundation, Maseru, Lesotho; ^2^ Elizabeth Glaser Pediatric AIDS Foundation, Washington, DC, United States; ^3^ Elizabeth Glaser Pediatric AIDS Foundation, Mbabane, Eswatini; ^4^ Department of International Health, Bloomberg School of Public Health, Johns Hopkins University, Baltimore, MD, United States

**Keywords:** PrEP, HIV, pregnant women, breastfeeding, postpartum, sub-Saharan Africa, prevention, medical records

## Abstract

**Introduction:**

Lesotho has reached epidemic control, PrEP is an important component in maintaining that and in reaching the goal of eliminating mother-to-child transmission.

**Methods:**

We conducted a retrospective review of existing, routine PrEP health records in 26 health facilities in Lesotho. PrEP visit data were collected for pregnant and postpartum women screened for PrEP and/or enrolled in PrEP programs from 1 January 2019 through 30 June 2021 with follow-up data collected up to the date of data abstraction per site between October 2021 and May 2022. Poisson regression with robust variance was used to evaluate the association between patient characteristics and continuation of PrEP.

**Results:**

Indications for starting PrEP were significantly associated with continuation in PrEP use. Women starting PrEP due to having a partner known to be living with HIV were the most likely to return for follow-up. In all age groups, the most common reason for starting PrEP was being in a serodiscordant relationship, though the proportion varies by age.

**Conclusion:**

As Lesotho is now in the process of optimizing PrEP use among pregnant and postpartum women, it is critical to revise data sources to capture information that will link PrEP records and ANC/PNC records and document pregnancy/postpartum status in order to better understand PrEP use and gaps in this population.

## 1. Introduction

Pregnancy and the postpartum period represent times of increased HIV acquisition risk (1). Driven by both biological and behavioral factors (2), this risk is further elevated in high-prevalence settings, such as sub-Saharan Africa (SSA) (3–5), which accounts for 70% of all new HIV infections globally (6). Compared to chronic infection, incident HIV during pregnancy and breastfeeding is associated with more than double the odds of vertical transmission in African cohorts (7). Breastfeeding is a particularly vulnerable period, with a study from Zimbabwe demonstrating a fourfold transmission increase in infants born to mothers with acute infection during breastfeeding (8, 9). Transmission during breastfeeding accounts for an estimated 50% of MTCT, with the proportion of transmission during breastfeeding increasing over time relative to intrauterine or intrapartum transmission (10). Overall, new infections after first antenatal care (ANC) account for a disproportionate number of infant infections (11, 12). Effective prevention strategies are urgently needed to reduce maternal and infant HIV acquisition.

Oral pre-exposure prophylaxis (PrEP) with daily tenofovir disoproxil fumarate (TDF) and emtricitabine (FTC) is an efficacious HIV prevention option for the reduction of vertical and horizontal transmission among HIV-negative pregnant and breastfeeding women in sub-Saharan Africa (SSA). Modeling estimates from South Africa indicate that widespread use of oral PrEP among pregnant and postpartum women could reduce vertical transmission by 41% and overall transmission by 2.5% (13). However, oral PrEP effectiveness and population-level prevention impact depend on the uptake and use in real-world implementation. The WHO extended guidance for the provision of PrEP to pregnant and breastfeeding women at substantial risk of HIV in 2017 (14) and there has been an expansion of oral PrEP in SSA, representing approximately one-third of PrEP prescriptions worldwide (15, 16). However, PrEP use globally, and utilization of PrEP by key groups such as pregnant and postpartum women, has failed to reach levels required to achieve the anticipated prevention impact or reduce vertical transmission (15, 16). Prevention with PrEP is user-controlled and empowers pregnant and breastfeeding women to make decisions regarding the prevention of HIV and gives them control over their HIV risks (13, 17–22). Extant research demonstrates limited uptake and continuation of PrEP among general users and pregnant and postpartum women (3, 23, 24). Research has identified key implementation considerations for PrEP success among pregnant and postpartum women including individual, social, and facility-level concerns such as the need for integration with antenatal and postnatal care (ANC/PNC), patient and provider education, stigma reduction, and person-centered health systems and guidelines supportive of screening, access, and ongoing support (15, 25–27). However, few studies in SSA have evaluated PrEP use among pregnant and postpartum women through routine health service provision. Understanding oral PrEP use may also inform the successful use of new HIV prevention technologies, such as injectable, long-acting Cabotegravir.

As high HIV prevalence countries in sub-Saharan Africa, including Lesotho, scale up the use of PrEP among pregnant and postpartum women within routine ANC/PNC, evidence regarding PrEP uptake and continuation in this population is essential to guide successful implementation. Lesotho has reached epidemic control (28); PrEP is an important component of maintaining that and getting to the goal of eliminating mother-to-child transmission. PrEP was first included in Lesotho national guidelines in April 2016 (29); however, there were no specific provisions for either the inclusion or exclusion of pregnant/poatpartum women until July 2019, when revised guidelines recommended routine screening for PrEP eligibility at ANC and PNC clinics (30). Using retrospective data abstracted from routine PrEP clients’ records at the health facilities in Lesotho, we sought to characterize the PrEP cascade and use patterns among pregnant and postpartum women to inform strategies to improve oral PrEP as an HIV prevention tool for women and their children.

## 2. Methods

### 2.1. Setting and study design

To understand real-world oral PrEP implementation and outcomes, we conducted a retrospective review of existing, routine PrEP health records in 26 health facilities run by the Government of Lesotho or the Christian Health Association of Lesotho. This included 6 hospitals and 20 health centers across four districts, all of which also received support from the Elizabeth Glaser Pediatric AIDS Foundation through the United States President’s Emergency Plan for AIDS Relief (PEPFAR). These health facilities included all medium-to-high PrEP patient volume sites in the four study districts and offered a range of HIV prevention, treatment, and maternal-child health (MCH) services (including ANC and PNC). Data abstraction at these facilities took place between October 2021 and May 2022.

At the time of data abstraction, PrEP was offered to clients meeting the following eligibility criteria: negative HIV test on the day of PrEP initiation; sexually active and at substantial risk of acquiring HIV infection (as determined by clinician screening or client request for PrEP); no suspicion of acute HIV infection; minimal risk of renal impairment; weight ≥35 kg, and willingness to use PrEP as prescribed. Following national guidelines, clients were asked to return 4 weeks and 8 weeks after PrEP initiation and then every 3 months thereafter for refills and assessment of adverse drug reactions, PrEP adherence, HIV risk, and HIV testing. Counseling and psychosocial support were available to clients at each visit as needed. National guidelines also recommend that PrEP refill visits for pregnant and postpartum women should coincide with ANC, PNC, or childhood immunization visits. All pregnant women in Lesotho are recommended to have at least eight antenatal visits, the first occurring as early as possible within 12 weeks of gestation (31). Postpartum care for the new mother and infants includes recommended visits within 6 h, 1, 6, 10, and 14 weeks, and 6 months post-delivery (31). Mothers living without HIV are counseled to exclusively breastfeed for the first 6 months then introduce complementary foods while continuing to breastfeed for 24 months or beyond. HIV testing is conducted every 3 months during the breastfeeding period (31).

### 2.2. Study participants

The study population included pregnant and postpartum individuals screened for PrEP and/or enrolled in PrEP programs from 1 January 2019 through 30 June 2021. Our data abstraction cohort included all individuals screened for or enrolled in PrEP. Because PrEP clinic records did not document pregnancy or postpartum status directly, we identified pregnant and postpartum as those with a documented PrEP entry point through ANC or PNC service points.

### 2.3. Data sources and data procedures

We abstracted individual-level screening, enrolment, and follow-up visit data from all PrEP-related routine forms at study sites, including PrEP risk and eligibility screening forms; PrEP-related registers; and individual client PrEP cards. For PrEP clients who seroconverted, we reviewed antiretroviral treatment (ART) registers and ART cards. PrEP follow-up visit data were collected up to the date of data abstraction per site between October 2021 and May 2022.

### 2.4. Statistical analysis

Our primary study outcome, continuation on PrEP, was measured dichotomously, defined as participants having any documented PrEP follow-up visit after PrEP initiation (yes/no). Patient age, marital status, and indications for starting PrEP were recorded directly from patient records. The study team classified each facility as urban or rural depending on the geographical location of each health facility and applied the Ministry of Health classification of sites as a hospital or health center. Documented screening for PrEP was measured as a dichotomous variable based on the presence or absence of a ‘PrEP Screening for Substantial Risk and Eligibility’ Form linked to a patient’s name or medical record number. PrEP start indications were taken from the PrEP card and grouped for analysis as pertaining to a serodiscordant relationship, multiple concurrent sexual partnerships, self-request, or other. According to the guidelines, clients who request PrEP should be initiated and provided with all information about the purpose of PrEP (30). Documentation of stopping PrEP and reasons for stopping PrEP were abstracted from the PrEP register.

We assessed the distribution of variables descriptively and used Poisson regression with robust variance to evaluate the univariate and multivariable association between patient characteristics and continuation of PrEP. Variable inclusion in our multivariable model was guided by statistical significance (*p* < 0.10 in unadjusted analyses) and applied theory of relationships between the variables based on past research (32, 33). We used multiple imputations with chained equations and 15 imputed data sets to account for missing covariate data in the multivariable model (34).

### 2.5. Ethical review

This study was approved by the Lesotho National Health Research Ethics Committee and Advarra Institution Review Board (IRB) in the United States of America. The protocol is limited to retrospective secondary analysis of data that is routinely documented as part of standard medical or program services. No additional patient information was collected outside of what is routinely recorded in patient records during standard medical care of patients. A waiver of consent was obtained from the IRB to abstract data from medical records. All study team members were trained in the protection of human subjects.

## 3. Results

**Figure 1:**
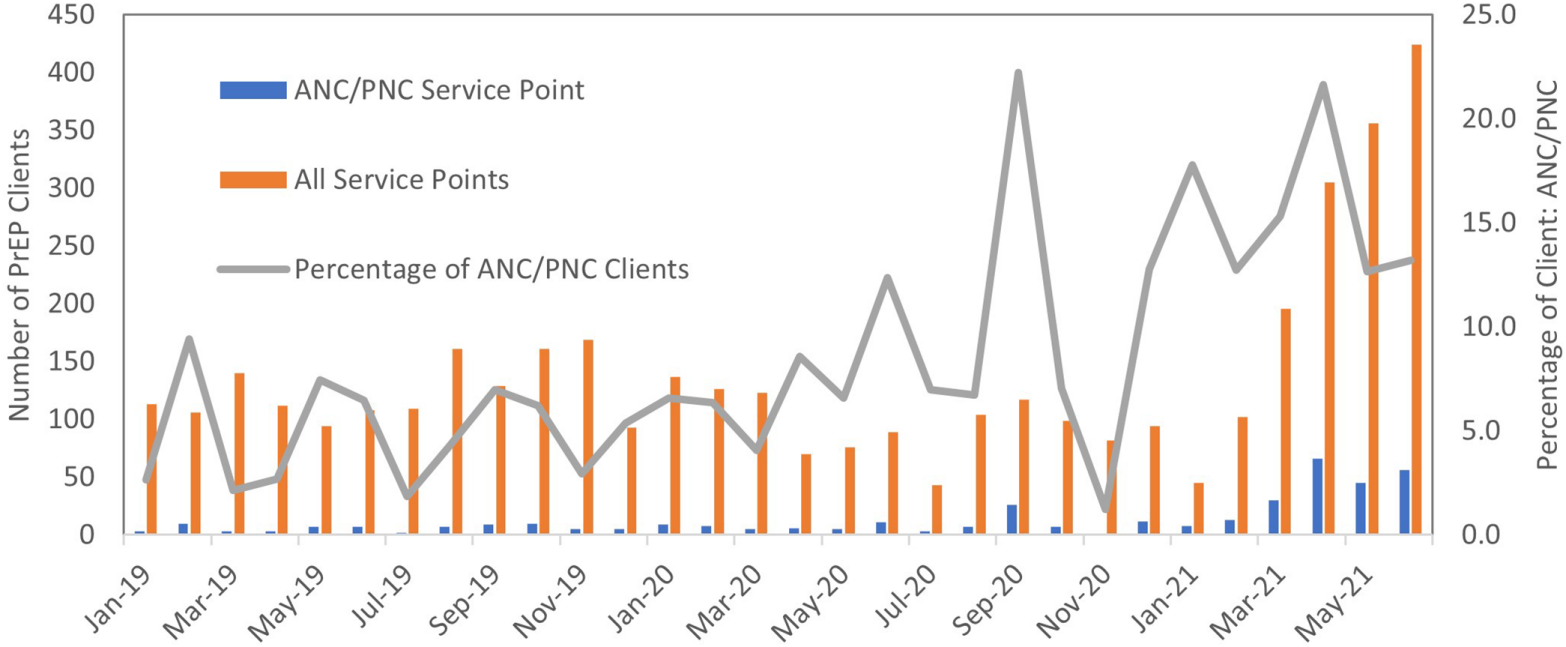
Distribution of PrEP clients by month of PrEP initiation.

A total of 4,098 participants from different service points in the health facilities were enrolled into the retrospective cohort. Among the 4,098 individuals screened for or enrolled in PrEP during our study period, we identified 389 (9%) pregnant or postpartum women from antenatal (ANC) and postnatal (PNC) service points initiated on PrEP. There was variation by site, with pregnant and postpartum women ranging from 0.3% to 17% of the total number of clients engaging in PrEP at study facilities. The proportion of clients initiated through ANC/PNC service points increased over time (Figure 1). ANC/PNC services were the most common entry point for younger female PrEP enrolees: 48% (*n* = 188) of female clients under age 25 screened for or enrolled in PrEP came from ANC/PNC services.

Data for pregnant and postpartum women were largely complete, with 17% missing indications for starting PrEP and 6% missing marital status. Table 1 details the demographic characteristics of the 389 pregnant and postpartum women. Women’s ages at PrEP initiation ranged from 14 to 48 years (median: 26 years). Among those with documented marital status (*n* = 364), 87% (*n* = 317) were married. The majority (76%, *n* = 295) attended ANC or PNC services at urban facilities. Nearly half (49%, *n* = 160) with a documented PrEP start indication were initiated due to being in a discordant relationship [most commonly a partner living with HIV who was not on antiretroviral treatment (ART) or was newly starting ART]. The second most common reason listed for initiating PrEP was the client reporting that either she—or more commonly her partner—had multiple concurrent partners (*N* = 82, 25%).

**Table 1:** Demographic characteristics^a^.

Characteristics	Total *N* = 389
Age (years)	
Median (IQR)	26 (21–31)
Range	14–48
Age categories	
14–20	114 (29.3)
21–34	222 (57.1)
35+	53 (13.6)
Marital Status	
Single/Divorced/Separated/Widowed	47 (12.9)
Married	317 (87.1)
Undocumented	25
Region	
Urban	295 (75.8)
Rural	94 (24.2)
Type of facility	
Hospitals	104 (26.7)
Health centers	285 (73.3)
Indications for starting PrEP (ungrouped)	
Participant self-requested PrEP	41 (12.7)
Serodiscordant relationship (not otherwise specified)	35 (10.8)
Serodiscordant relationship: partner not on ART or on ART < 12 months	76 (23.4)
Serodiscordant relationship: partner known to have elevated viral load (>1,000 copies/ml) and/or poor adherence	48 (14.8)
Serodiscordant relationship: partner not on ART or on ART < 12 months, AND partner known to have elevated viral load and/or poor adherence	1 (0.3)
Has multiple concurrent sexual partners	6 (1.9)
Client believes her partner has multiple concurrent sexual partners	76 (23.4)
Unknown partner HIV status	23 (7.1)
Patient being in antenatal or postnatal care only documented indication for PrEP start	9 (2.8)
Frequent exposure	3 (0.9)
Individual at high risk of being forced to have sex	6 (1.9)
Undocumented	65
Indications for starting PrEP (grouped)	
Self-requested PrEP	41 (12.7)
Serodiscordant relationship	160 (49.3)
Multiple concurrent partners	82 (25.3)
Other	41 (12.7)
Undocumented	65

^a^ Percentages are among participants with data for that variable (Documented).

**Figure 2:**
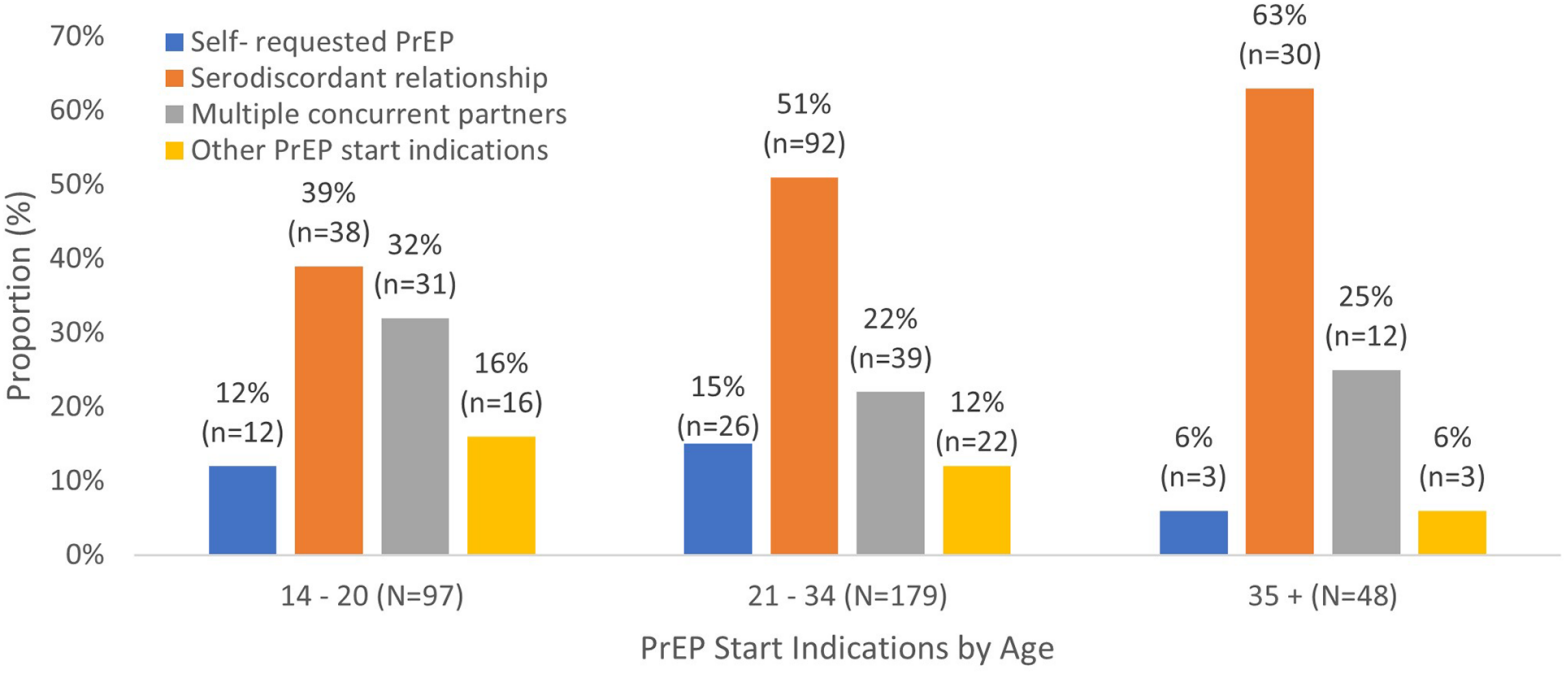
Distribution of PrEP start indications by Age.

Figure 2 shows the distribution of PrEP start indications by age among those with documented start indications. In all age groups, the most common reason for starting PrEP was being in a serodiscordant relationship, though the proportion varies by age. Serodiscordant relationships account for 63% of PrEP initiations among women aged 35 and older compared to 39% of initiations among women aged 14–20 years.

Data on PrEP screening were limited; only 245 (63%) of women initiating PrEP had documentation of screening. There were no records of pregnant or postpartum women being screened and not initiating PrEP. Having documented screening (i.e., a Screening Form linked to your name or medical record number) was more common among women in urban facilities compared to rural facilities (69% vs. 45%, *p* < 0.001) and in health centers compared to hospitals (71% vs. 40%, *p* < 0.001). Five sites had no completed screening forms for pregnant or postpartum women while three sites had screening forms available for all pregnant/postpartum women. Ninety-one percent of women with a screening form reported at least one behavioral risk factor for HIV acquisition: 17% (*n* = 38) reported unprotected sex in the last 3 days with someone living with HIV who was not on treatment and 39% (*n* = 87) had condom-less sex or other high-risk HIV exposure in the past 2–6 weeks.

Of the 389 pregnant and postpartum women initiated on PrEP, 40% (*n* = 156) had no recorded follow-up visits, 76 (20%) had only one recorded follow-up visit post-PrEP initiation, and the remaining 40% (*n* = 157) had at least two documented follow-up visits (the maximum number of documented follow-up visits was 14). Table 2 presents the univariate and multivariable analysis findings related to factors associated with the continuation of PrEP (i.e., having any documented follow-up visit after PrEP initiation). Having any recorded follow-up after PrEP initiation was significantly associated with initiating PrEP at an urban facility compared to a rural facility [adjusted prevalence ratio (aPR) = 1.34, 95% CI = (1.07; 1.67)]. Women who started PrEP due to serodiscordant relationships [aPR = 2.13; 95% CI = (1.38; 3.29)] or who started due to multiple concurrent partnerships [aPR = 1.78; 95% CI = (1.14; 2.77)] were more likely to continue using PrEP than women who self-requested PrEP (*p *≤ 0.01). Neither age nor marital status were significantly associated with continuation.

**Table 2:** Factors associated with documentation of any follow-up visit after PrEP initiation.

			Analysis based on Multiple Imputation			
Characteristics	*n* (%)	Total	Unadjusted		Adjusted	
Continuation: Any follow-up after initiation	233 (59.9)	389	PR (95% CI)	*p*-value	PR (95% CI)	*p*-value
Age groups						
Less 21	63 (55.3)	114	1	0.379	1	0.942
21–34	135 (60.8)	222	1.10 (0.90; 1.34)		1.01 (0.83; 1.23)	
35+	35 (66.0)	53	1.19 (0.93; 1.54)		1.04 (0.81; 1.35)	
Marital Status						
Single/Divorced/Separated/Widowed	25 (53.2)	47	1	0.354	1	0.971
Married	193 (60.9)	317	1.14 (0.86; 1.52)		1.01 (0.75; 1.35)	
Region						
Rural	46 (48.9)	94	1	0.024	1	0.011
Urban	187 (63.4)	295	1.30 (1.04; 1.62)		1.34 (1.07; 1.67)	
Type of facility						
Hospitals	68 (65.4)	104	1	0.165		
Health Centers	165 (57.9)	285	0.89 (0.75; 1.05)			
Indications for starting PrEP						
Self-requested PrEP	14 (34.2)	41	1	0.001	1	0.002
Serodiscordant/discordant couples	112 (70.0)	160	2.04 (1.32; 3.14)		2.13 (1.38; 3.29)	
Multiple concurrent partners	46 (56.1)	82	1.64 (1.05; 2.57)		1.78 (1.14; 2.77)	
Other	20 (48.8)	41	1.40 (0.83; 2.37)		1.55 (0.91; 2.62)	

Only six women had documentation of stopping PrEP: four due to patient decision, and two due to HIV seroconversion. The two participants who had documented HIV seroconversion; were both initiated on ART.

## 4. Discussion

We assessed screening, initiation, and continuation of oral PrEP among pregnant and postpartum women accessing care through public health facilities in Lesotho to understand real-world PrEP outcomes and inform interventions to improve HIV prevention. Indications for starting PrEP were significantly associated with continuation in our study. Women starting PrEP due to having a partner known to be living with HIV were the most likely to return for any follow-up. While these women may be more likely to have continued elevated HIV risk over time, it is also possible that having a partner living with HIV may have reduced stigma or fears around taking PrEP in the home. Rural facilities had lower rates of PrEP continuation, underscoring the need for differentiated models of service delivery (including community-based distribution and multi-month PrEP dispensing) to ensure that difficulties in accessing sites in rural areas are not prohibitive to PrEP continuation.

Our findings underscore the need to promote and expand the uptake of PrEP among pregnant and postpartum women in Lesotho. Despite utilizing healthcare services at higher rates than the general adult population, pregnant and postpartum women represented a minority (9.5%) of PrEP initiations during this time period (though there was evidence of an increased trend over time in both the number of pregnant and postpartum women initiated and the proportion of PrEP initiations coming from ANC/PNC services). ANC/PNC services remain a critical means of reaching younger women, who are at increased risk of HIV. With guidelines revised in 2022 to include universal screening of pregnant and postpartum women living without HIV for PrEP eligibility, it will be important to evaluate whether there was a subsequent continued increase not just in the number of pregnant and postpartum women screened and enrolled in PrEP but also in the proportion of pregnant and postpartum women within the total cohort of PrEP clients. This evaluation will only be possible with improved routine documentation of screening and eligibility for PrEP within health facilities, which is a significant limitation of our study. This documentation is important not only to understand whether PrEP screening and initiation are being conducted in accordance with the guidelines but also to understand the true PrEP refusal rate.

A number of strategies have been documented to promote PrEP uptake among cisgender women (20, 22, 35). Differentiated models of PrEP delivery including client-centered approaches, offering multiple options for PrEP (including longer-acting drugs), provision of PrEP information through peer educators, and tailored PrEP education and messaging have been identified as facilitators to PrEP uptake and adherence (20, 22, 26, 35, 36). However, gaps still exist in the provision of PrEP to pregnant and postpartum women, including scale-up and integration of PrEP into routine antenatal and postnatal clinics (4, 25, 26).

Our findings are consistent with a number of other studies showing low levels of continuation of PrEP, including among pregnant and postpartum women (3, 24, 27, 37). Other studies with women living with HIV have also found sub-optimal adherence to ART refills during the post-partum period (38–41). However, these data are difficult to interpret without reliable data on the risk for HIV acquisition following PrEP initiation. For example, we cannot assess how many women may be discontinuing PrEP due to reduced risk (including women seeking event-driven PrEP around holidays when partners living with HIV return from remote work, which is common in Lesotho, or cultural practices around sexual activity during pregnancy or postpartum). Other women may have transferred their care to another facility; as there was no active tracking or outreach to women who did not return for PrEP refills, this would not have been captured. Understanding and documenting fluctuations in HIV risk, as well as a better understanding of the motivation to adhere to PrEP, will be even more critical as countries like Lesotho introduce long-acting cabotegravir (CAB-LA) as an option for HIV prevention. The high proportion of women in our study who did not return for any follow-up PrEP visits or refills (coupled with the low documented rate of PrEP refusal) may indicate that some women accepted PrEP at the recommendation of their providers despite low motivation to begin or continue taking PrEP. While CAB-LA offers a number of benefits compared to oral PrEP, low motivation to continue on PrEP would be very concerning given the increased risk of integrase inhibitor resistance associated with HIV acquisition while recently or currently on cabotegravir-PrEP. Motivation to continue PrEP among postpartum women may have also changed over time as concerns about mother-to-child transmission decreased after delivery and breastfeeding cessation.

There are other individual, social, and facility-related factors that could influence PrEP continuation that are not captured in available routine data. Pill fatigue, low awareness of optimal PrEP dosing, misalignment of HIV risk perception versus actual risk, concerns about side effects, forgetting to take PrEP daily, stigma associated with using antiretrovirals for prevention, gender norms, financial constraints, and accessibility of health facilities are some of the barriers that have been shown to undermine full utilization of PrEP among pregnant and postpartum women (19, 20, 22, 24, 35). Further studies with patients and healthcare workers are necessary to address this gap and consider which, if any, data points should be added to routine PrEP data collection.

Utilization of real-world program data is critical to understand real-world implementation. Our study identified key gaps in routine data that, if improved, may support improved service provision. While appropriate screening is considered critical to improving oral PrEP prevention impact (i.e., identifying women who can benefit and enrolling them), screening data were unavailable for 37% of our cohort. While lack of documented screening was not a barrier to initiation among these 37%, consistent documentation of screening is critical to ensuring appropriate PrEP use. Additionally, there were no records of women being screened but identified to be ineligible or choosing not to initiate. Improved documentation of all individuals screened is necessary to understand: 1. who is being screened, 2. what proportion of pregnant and postpartum people are ineligible, and 3. what proportion of those eligible refuse PrEP. Programmatic assessment identified limited availability of and inconsistent knowledge about screening forms as a barrier to utilization. Support for improved documentation is recommended to ensure optimized PrEP service delivery. Further, while adherence was measured in routine records, inconsistent recording of adherence, mixing days adherence/7 days, and % of pills taken made assessment through routine record review infeasible. As understanding adherence within routine health settings is critical to assessing prevention-effective use, improving routine data collection will be important.

As with any study relying on routine data (an important source for implementation science and program improvement efforts), our study is limited by incomplete data. In addition, routine PrEP-related documentation did not directly capture whether a woman was currently pregnant or postpartum; as a result, we may have excluded a number of pregnant and postpartum women from analysis if they were screened or enrolled in PrEP outside of the ANC/PNC clinics.

## 5. Conclusion

As Lesotho is now in the process of optimizing PrEP use among pregnant and postpartum women, it is critical to revise data sources to capture information that will link PrEP records and ANC/PNC records and document pregnancy/postpartum status in order to better understand PrEP use and gaps in this population.

## Data Availability

The datasets presented in this article are not readily available as they are based on abstraction from routine health records owned by the Lesotho Ministry of Health, who must approve any additional use of the data. To request data access, contact corresponding author with details on the intended use of the data. Requests to access the datasets should be directed to lmasenyetse@pedaids.org.
